# Advances in cardiovascular supplementation: mechanisms, efficacy, and clinical perspectives

**DOI:** 10.3389/fmolb.2025.1699492

**Published:** 2026-01-26

**Authors:** Xun Wu, Tanwei Fang

**Affiliations:** 1 The Second Department of Thoracic Surgery, Hunan Cancer Hospital and The Affiliated Cancer Hospital of Xiangya School of Medicine, Central South University, Changsha, Hunan, China; 2 Hunan Provincial Chest Hospital, Tuberculosis Control Institution of Hunan Province, Changsha, Hunan, China

**Keywords:** arrhythmia, dyslipidemia, heart failure, meta-analysis, nutritional status, precision medicine

## Abstract

The integration of nutritional supplementation into cardiovascular disease (CVD) prevention and management represents a dynamic and often contentious field. Moving beyond traditional paradigms, recent research has sought to elucidate the precise molecular mechanisms, establish robust clinical efficacy through large-scale trials, and identify specific patient populations that may derive the greatest benefit. This review synthesizes the current evidence on key supplements—including omega-3 fatty acids, coenzyme Q10 (CoQ10), magnesium, and selenium—evaluating their roles from biochemical, translational, and clinical viewpoints. We explore the conditions under which certain supplements have transitioned from general wellness products to targeted therapeutic adjuvants, address the controversies surrounding their use, and discuss future directions for research and clinical application.

## 1 Introduction

Cardiovascular disease (CVD) is the leading cause of mortality worldwide, accounting for nearly one-third of global deaths each year (Roth et al., 2020). Despite major advancements in pharmacotherapies and interventional cardiology, substantial residual risk persists among patients with established disease (Ganda et al., 2018). While lifestyle modifications (e.g., smoking cessation, regular physical activity, and heart-healthy diets low in salt and saturated fats) and pharmacological treatment remain the bedrock of CVD management, increasing attention has turned to nutraceuticals and bioactive dietary supplements as complementary strategies (Visseren et al., 2021).

The appeal of these agents stems from their capacity to modulate key pathophysiological contributors in atherosclerosis and heart failure, including oxidative stress, chronic vascular inflammation, endothelial dysfunction, dyslipidemia, and mitochondrial energy deficits (Virani et al., 2021). Additionally, many nutraceuticals are perceived to possess favorable safety and tolerability profiles compared to conventional medications, making them attractive for long-term risk reduction (Santini et al., 2018).

However, the field is marked by heterogeneous and sometimes contradictory evidence. Discrepancies in trial design, supplement formulation, purity, dose, treatment duration, and baseline nutritional status have all contributed to inconsistent findings (Banach et al., 2018; Cicero et al., 2017). For instance, although certain supplements (e.g., omega-3 fatty acids and coenzyme Q10 [CoQ10]) have demonstrated cardiovascular benefits in robust randomized controlled trials, other popular agents (e.g., vitamin E and beta-carotene) have failed to show efficacy or have even indicated possible harm in large-scale studies (Sesso et al., 2008).

**FIGURE 1:**
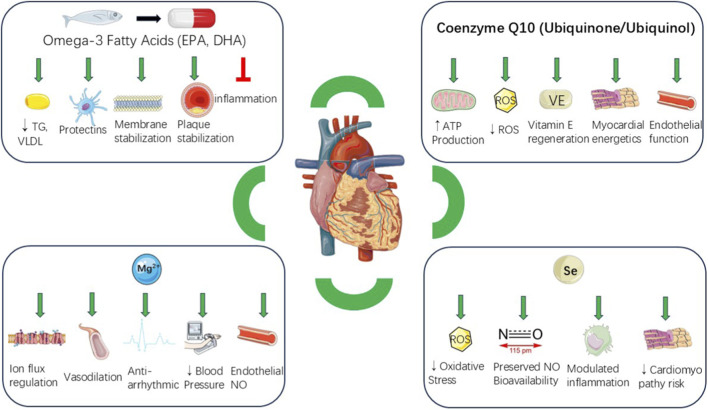
Cardioprotective mechanisms of key nutritional supplements in cardiovascular disease. Schematic representation of the major biological pathways through which nutritional supplementation supports cardiovascular protection.

More recently, mechanistic precision and personalized approaches have come to the forefront. Advances in metabolomics and nutrigenomics suggest that specific patient subpopulations may realize the greatest benefit, indicating a move away from generalized supplementation toward a more targeted adjunctive therapy integrated with evidence-based cardiovascular care (Minihane et al., 2015). This review evaluates the mechanisms of action and recent advances in cardiovascular supplementation (Figure 1), distinguishing well-established clinical evidence from hypothesis-generating findings, and highlights emergent areas where nutraceuticals may reshape preventive and therapeutic cardiology.

## 2 Omega-3 fatty acids

### 2.1 Mechanisms of action

Omega-3 polyunsaturated fatty acids, primarily eicosapentaenoic acid (EPA) and docosahexaenoic acid (DHA), have been extensively studied for their cardioprotective properties. Their benefits are mediated via multiple mechanisms:

1. Triglyceride Reduction. EPA and DHA markedly decrease hepatic very-low- density lipoprotein (VLDL) synthesis and improve triglyceride clearance (Khan et al., 2021).
2. Anti-Inflammatory Effects. They serve as substrates for specialized pro-resolving mediators, such as resolvins and protectins, which actively resolve inflammation, a key driver of atherosclerosis (Khan et al., 2021).
3. Anti-Arrhythmic Properties. By stabilizing cardiac myocyte membranes, these fatty acids reduce susceptibility to fatal ventricular arrhythmias (Nicholls et al., 2020).
4. Plaque Stabilization. They may bolster endothelial function and diminish the rupture propensity of atherosclerotic plaques (Budoff et al., 2020).

### 2.2 Clinical evidence and trials

The clinical evidence for omega-3 fatty acids has evolved significantly. Early trials, such as JELIS, showed that high-purity eicosapentaenoic acid reduced major coronary events among statin-treated patients. The more recent REDUCE-IT trial (n = 8,179) found that 4 g/day of icosapent ethyl (a purified eicosapentaenoic acid ethyl ester) significantly reduced cardiovascular death (HR 0.75, 95% CI 0.68–0.83), myocardial infarction, and stroke in high-risk individuals with elevated triglycerides despite statin therapy. Conversely, the STRENGTH trial (n = 13,078)—using a carboxylic acid formulation of eicosapentaenoic acid plus docosahexaenoic acid—was terminated prematurely for futility (HR 0.99, 95% CI 0.90–1.09) (Nicholls et al., 2020).

These results suggest that not all omega-3 formulations are equivalent. Contemporary meta-analyses indicate that eicosapentaenoic acid-only therapy yields greater cardiovascular benefits than combinations of eicosapentaenoic acid and docosahexaenoic acid, despite comparable triglyceride reductions (Khan et al., 2021). Furthermore, imaging studies, such as EVAPORATE, suggest that icosapent ethyl can slow or even reverse coronary plaque progression, pointing to pleiotropic actions beyond triglyceride lowering (Budoff et al., 2020).

### 2.3 Practical considerations

The clinical application of high-dose omega-3 fatty acids is now guided by specific evidence and safety considerations.

Formulation and Dose: The substantial cardiovascular benefits demonstrated in REDUCE-IT are attributed to high-dose (4 g/day), purified eicosapentaenoic acid (icosapent ethyl). This effect does not appear to be a class effect of all omega-3 formulations.

Safety Advisory: A dose-dependent increase in the risk of atrial fibrillation has been consistently observed in major trials, especially at the 4 g/day dose, prompting regulatory safety advisories (Gencer et al., 2021).

Guideline Recommendations: Based on this evidence, major guidelines now endorse the selective use of icosapent ethyl. It is typically assigned a Class IIb recommendation for statin-treated patients with persistent hypertriglyceridemia (triglycerides of 150–499 mg/dL) and adequately controlled low-density lipoprotein cholesterol (<100 mg/dL) (Virani et al., 2023).

## 3 Coenzyme Q10 (CoQ10) and ubiquinol

Coenzyme Q10 (CoQ10), present in oxidized (ubiquinone) and reduced (ubiquinol) forms, is integral to the mitochondrial respiratory chain and serves as a lipid-soluble antioxidant (Raizner and Quiñones, 2021). Beyond its primary function in ATP generation, CoQ10 exerts antioxidant and redox-modulating effects, including vitamin E regeneration and reduced lipid peroxidation, thus preserving mitochondrial health (Raizner and Quiñones, 2021). An emerging body of evidence, including a recent umbrella review, underscores its broader potential in improving metabolic parameters, such as fasting blood glucose and insulin resistance, which are crucial in cardiovascular health (Patiño-Cardona et al., 2024). These mechanisms are particularly relevant in the myocardium, given its high energy requirements and susceptibility to oxidative stress.

### 3.1 Mechanistic of action

1. Energy Enhancement. Through facilitation of electron transport across mitochondrial complexes, CoQ10 supplementation can improve myocardial bioenergetics, particularly in systolic dysfunction (Mortensen et al., 2014).
2. Antioxidant Activity. Ubiquinol scavenges reactive oxygen species and assists in vitamin E regeneration, thereby mitigating oxidative damage and inflammatory processes (Siti et al., 2015).
3. Statin Side-Effect Mitigation. Statins inhibit the mevalonate pathway, lowering endogenous CoQ10 levels, which may contribute to statin-associated muscle symptoms (SAMS) (Kawashima et al., 2020).

### 3.2 Clinical evidence in heart failure

The Q-SYMBIO trial (n = 420)—administering 300 mg/day of CoQ10—demonstrated improvements in New York Heart Association (NYHA) class, reductions in major adverse cardiovascular events (MACE) (HR 0.50, 95% CI 0.32–0.80), and lower all-cause mortality in patients with moderate-to-severe heart failure (Mortensen et al., 2014). A 2024 meta-analysis (pooled n = 2,350) of 33 randomized controlled trials concluded that Coenzyme Q10 supplementation significantly reduced mortality (relative risk 0.64) and hospitalizations for heart failure (relative risk 0.50), while improving left ventricular ejection fraction, B-type natriuretic peptide levels, and 6-min walk distance (Xu et al., 2024). This positive signal is further corroborated by another recent umbrella review, which highlighted that Coenzyme Q10 consistently demonstrates beneficial effects on cardiac function and clinical outcomes across meta-analyses (Alarcón-Vieco et al., 2023). Nonetheless, the overall evidence quality remains moderate, partly due to heterogeneity in dosing and trial design.

### 3.3 Emerging data in heart failure with preserved ejection fraction and endothelial function

A 2022 phase II, double-blind, randomized trial in patients with preserved ejection fraction (HFpEF) reported that ubiquinol (600 mg/day), alone or combined with D-ribose, modestly enhanced LVEF and alleviated symptom burden, though exercise capacity improvements were inconsistent (Pierce et al., 2022). A crossover pilot study in heart failure with reduced ejection fraction demonstrated that ubiquinol supplementation could improve endothelial function, as measured by peripheral arterial tonometry (Kawashima et al., 2020). Consistent with these findings, a 2024 systematic review showed that Coenzyme Q10 contributes to improved flow-mediated dilation (FMD), indicating broader vascular benefits beyond myocardial energetics (Daei et al., 2024).

### 3.4 Statin-associated myopathy

Although there is mechanistic plausibility for Coenzyme Q10 in alleviating SAMS, a 2021 meta-analysis identified no significant improvements in myalgia or creatine kinase levels relative to placebo (Wei et al., 2022). Likewise, a 2022 randomized trial documented higher intramuscular Coenzyme Q10 concentrations but no consistent clinical benefit (Dohlmann et al., 2022). Hence, routine Coenzyme Q10 supplementation for SAMS is not currently indicated, though select patients may experience subjective relief.

### 3.5 Practical considerations

Formulation significantly influences Coenzyme Q10 bioavailability; lipid-based and solubilized preparations generally achieve superior absorption. Both ubiquinone and ubiquinol interconvert *in vivo*, although ubiquinol may yield higher plasma levels under some conditions (Fladerer and Grollitsch, 2023). Typical doses in cardiovascular research range from 100 to 400 mg/day and are well tolerated, except for occasional gastrointestinal effects. Interaction with warfarin (due to Coenzyme Q10’s structural similarity to vitamin K) necessitates monitoring of the international normalized ratio (INR) (Raizner and Quiñones, 2021). Overall, Coenzyme Q10—especially ubiquinol—holds promise as an adjunct therapy in heart failure management, although larger, high-quality trials are warranted to refine recommendations, particularly regarding statin-associated muscle symptoms.

## 4 Magnesium

Magnesium serves as a cofactor in over 300 enzymatic reactions that govern vascular tone, cardiac excitability, glucose–insulin dynamics, and energy metabolism (AlShanableh and Ray, 2024; Nielsen, 2024). Insufficient magnesium disrupts electrophysiological stability and endothelial biology and is epidemiologically associated with elevated cardiometabolic risk.

### 4.1 Mechanistic pathways

1. Vasodilation. Magnesium ions compete with calcium ions at L-type channels, modulating vascular smooth muscle contractility and reducing peripheral resistance (Nielsen, 2024).
2. Anti-Arrhythmic Effects. By stabilizing sarcolemmal ion flux and atrioventricularnodal conduction, magnesium elevates the threshold for early afterdepolarizations, playing a potential role in controlling torsades de pointes and atrial fibrillation triggers (AlShanableh and Ray, 2024; Nielsen, 2024).
3. Endothelial Function. Magnesium supports nitric oxide (NO) bioavailability and restrains oxidative stress; low magnesium can induce a pro-inflammatory, pro-atherogenic endothelial environment (Janaszak-Jasiecka et al., 2023). These findings suggest a biological rationale for targeted supplementation in deficient individuals rather than indiscriminate use.

### 4.2 Deficiency and cardiometabolic associations

Population-based data (NHANES 1999–2020) indicate persistent magnesium intake shortfalls, with specific demographics particularly affected (Yan et al., 2024). Low serum magnesium levels correlate with insulin resistance and poorer outcomes in type 2 diabetes, as well as increased atrial and ventricular arrhythmias (Oost et al., 2023; Rooney et al., 2020). These insights align with intervention studies that explore magnesium as a “test-and-treat” approach.

### 4.3 Blood pressure effects

A 2024 umbrella meta-analysis (n = 2,008) of randomized controlled trials found that magnesium supplementation lowers blood pressure, although effects were modest and varied by baseline status, dose, and treatment duration (Alharran et al., 2024). Another 2024 meta-analysis reported a 2–3 mmHg decrement in systolic blood pressure, with more pronounced effects observed in individuals with suboptimal magnesium status, hypertension, or higher baseline blood pressure, and with doses exceeding 300 mg/day over a prolonged duration (≥8 weeks) (Behers et al., 2024).

### 4.4 Endohelial function

Preliminary mechanistic studies suggest magnesium may improve surrogate vascular endpoints. In a randomized crossover trial of healthy adults, 1 week of magnesium supplementation enhanced brachial artery flow-mediated dilation, while *in vitro* research shows that low-magnesium conditions downregulate endothelial NO pathways and upregulate pro-oxidant signaling (Byrne et al., 2021). Larger, well-designed trials are needed to substantiate these findings.

### 4.5 Arrhythmias and acute indications

Intravenous magnesium sulfate remains a guideline-endorsed treatment for torsades de pointes and is part of the Advanced Cardiovascular Life Support protocol; however, routine use in undifferentiated cardiac arrest is not recommended (Perman et al., 2024). Magnesium sulfate is also standard care in eclampsia for seizure prevention and management (Author Anonymous, 2020). In atrial fibrillation, evidence is context-dependent: a 2021 meta-analysis found adjunctive IV magnesium more effective at rate control than rhythm conversion in non-postoperative atrial fibrillation, while a 2022 cohort study reported enhancements in spontaneous cardioversion when potassium and magnesium were co-administered (Ramesh et al., 2021; Osawa et al., 2020; Curran et al., 2023). For postoperative atrial fibrillation in cardiothoracic patients, low-dose or continuous magnesium infusion can be beneficial, although results are inconsistent in non-cardiac surgery settings.

### 4.6 Practical considerations

Current consensus supports targeting magnesium replacement in individuals with documented hypomagnesemia or specific arrhythmogenic risks (e.g., prolonged QT interval). Oral formulations with higher bioavailability (e.g., magnesium citrate, glycinate) are generally preferred for chronic supplementation. Clinicians should monitor for gastrointestinal intolerance (particularly with oxide or chloride salts) and the risk of hypermagnesemia—particularly in patients with chronic kidney disease (Van Laecke, 2024).

## 5 Selenium

### 5.1 Mechanisms of action and biological functions

Selenium is a trace element incorporated into selenoproteins (e.g., glutathione peroxidases, thioredoxin reductases) that maintain redox homeostasis and mitigate oxidative damage (Zagrodzki and Laszczyk, 2006; Rayman, 2012). Through these antioxidant and anti-inflammatory functions, selenium influences vascular tone, preserves nitric oxide (NO) bioavailability, and modulates endothelial dysfunction (Papp et al., 2010). Selenoprotein P (SELENOP), the principal selenium transporter, serves as a functional biomarker of systemic selenium status and a predictor of cardiovascular risk (Schöttker et al., 2024).

### 5.2 Deficiency states and historical perspective

The pivotal role of selenium in cardiovascular integrity is historically underscored by its ability to prevent Keshan disease, a once-endemic dilated cardiomyopathy in parts of China with profoundly selenium-deficient soil (Li et al., 2024; Zhou et al., 2018). This landmark finding underscores selenium’s pivotal role in cardiovascular integrity. Conversely, in selenium-replete populations, supraphysiologic dosing yields inconsistent outcomes and has not reliably demonstrated cardioprotective benefit.

### 5.3 Clinical evidence and status-dependent effects

Recent data support a U- or L-shaped association between selenium status and cardiorenal outcomes. In the ESTHER study (n = 7,186; 17-year follow-up), low SELENOP levels correlated with higher all-cause and cardiovascular mortality, particularly in the lowest tertile (Schöttker et al., 2024). Analysis of NHANES 2007–2018 (n = 25,801) suggests that moderate selenium intake correlates with reduced CVD prevalence and mortality, whereas higher intakes show diminished or no added benefit (Zhang et al., 2024). Functional studies indicate that glutathione peroxidase activity saturates at 70–90 μg/L serum selenium, further explaining why supplementation beyond this threshold may not enhance antioxidant capacity (Brodin et al., 2020).

### 5.4 Practical considerations

In selenium-replete populations, large trials (e.g., SELECT) found no reduction in cardiovascular events with 200 μg/day of selenomethionine and even observed a potential rise in type 2 diabetes incidence (Zhang et al., 2017). More recently, SUSTAIN-CSX (n = 1,416, cardiac surgery patients) showed no improvement in postoperative organ dysfunction or mortality with high-dose IV sodium selenite (Stoppe et al., 2023). Meta-analyses consistently indicate no overall cardiovascular advantage from selenium supplementation in replete cohorts (Ouyang et al., 2022). Nonetheless, certain vulnerable groups, including those with chronic kidney disease, may benefit from targeted repletion (Zhu et al., 2023). Safety recommendations vary geographically, with the European Food Safety Authority setting the tolerable upper intake level (UL) at 255 μg/day, while the U.S. UL stands at 400 μg/day (Turck et al., 2023). Clinical consensus favors individualized, biomarker-based supplementation in genuinely deficient individuals.

## 6 Other noteworthy supplements

### 6.1 Vitamin D

Although vitamin D exerts immunomodulatory and renin–angiotensin influences relevant to vascular biology, randomized trials in generally replete populations have not demonstrated significant reductions in major cardiovascular outcomes. In the Australian D-Health RCT (n = 21,315; 60,000 IU/month cholecalciferol), vitamin D supplementation led to a non-significant 9% reduction in major adverse cardiovascular events (MACE), driven primarily by myocardial infarction, while stroke outcomes remained unchanged (Cheng et al., 2025). A 2025 meta-analysis similarly reported no significant decrease in MACE (pooled hazard ratio ∼0.96) (Thompson et al., 2023). Earlier publications also revealed no clear cardiovascular benefit in unselected individuals (Pei et al., 2022). Consequently, deficiency correction remains appropriate for high-risk groups, but routine supplementation for primary cardiovascular prevention is not currently recommended (Musazadeh et al., 2023).

### 6.2 L-carnitine

L-Carnitine supports mitochondrial fatty acid transport, and meta-analyses have shown modest lipid-lowering effects (e.g., reductions in LDL-C and triglycerides) at doses exceeding 2 g/day (Musazadeh et al., 2023). Although a 2021 Cochrane review suggested improvements in intermittent claudication with propionyl-L-carnitine, the evidence base is dated and lacks adequately powered contemporary trials (Kamoen et al., 2021). Further caution arises from a 2022 Mendelian randomization study linking higher genetically proxied carnitine to increased coronary artery disease and heart failure risks (Zhao et al., 2022). Thus, clinicians should weigh potential lipid-related benefits against emerging safety considerations.

### 6.3 Garlic extract

Putative benefits include vasodilation via enhanced NO bioavailability, possible ACE inhibition, and antioxidant properties. Recent randomized trials and meta-analyses indicate modest reductions in blood pressure and minor improvements in lipid profiles when using standardized aged or freeze-dried garlic extracts (Saadh et al., 2024; Rahmatinia et al., 2024; Du et al., 2024). Although these effects may be clinically relevant in specific subsets (e.g., mildly hypertensive or dyslipidemic patients), overall gains appear moderate compared to conventional therapies.

## 7 Discussion

The evolving landscape of cardiovascular supplementation reflects a paradigm shift from empirical, population-wide approaches toward precision-based, biomarker-guided strategies. Traditional supplementation practices, often characterized by heterogeneous results and variable clinical utility, are increasingly being replaced by evidence-informed frameworks integrating molecular insights, nutrigenomics, and translational trial design. This shift is underscored by recent findings that highlight the central role of context-dependent efficacy, whereby baseline nutrient status, genetic variability, and comorbid metabolic profiles critically determine clinical outcomes (Butler et al., 2020). To provide a clearer comparison, we summarized key meta-analyses in Table 1. This structured overview highlights the highest level of available evidence on efficacy and safety, guiding clinicians toward evidence-based decision-making.

**TABLE 1:** Summary of key meta-analyses on cardiovascular nutritional supplementation.

Supplement	Reference (year)	Population	Sample size (n)	Key outcomes
Omega-3 fatty acids (EPA)	Aung et al., JAMA Cardiol (2024) [9]	High CV risk, on statins	13,078	Significant reduction in MACE with EPA-only formulations
Omega-3 fatty acids (EPA + DHA)	Abdelhamid et al., Cochrane Database Syst Rev (2022)	General and high-risk populations	>100,000	Little to no effect on major CV events or mortality with combined EPA + DHA.
Coenzyme Q10	Jiang et al., Eur J Heart Fail (2024) [18]	Heart failure patients	2,350	Significant reduction in all-cause mortality and HF hospitalization; improvement in LVEF.
Magnesium	Zhang et al., Eur J Nutr (2024) [33]	Adults, primarily hypertensive	2,008	Modest reduction in systolic BP (−2 to −3 mmHg), effect modified by baseline status and dose
Selenium	Vinceti et al., Cochrane Database Syst Rev (2023) [51]	Selenium-replete populations	>40,000	No beneficial effect on CV outcomes; potential increase in type 2 diabetes risk

• MACE: major adverse cardiovascular events.

• CV: cardiovascular.

• HF: heart failure.

• LVEF: left ventricular ejection fraction.

• BP: blood pressure.

The paradigm of “one-size-fits-all” supplementation is increasingly untenable. Biomarker-driven stratification tools, including the omega-3 index, serum magnesium levels, circulating selenoprotein P, and 25-hydroxyvitamin D, are emerging as critical determinants of therapeutic efficacy. For instance, icosapent ethyl (EPA-only therapy) has demonstrated consistent reductions in major adverse cardiovascular events in hypertriglyceridemic patients, whereas mixed EPA + DHA formulations have yielded inconsistent results (Bhatt et al., 2019; Rizos et al., 2021). Similarly, selenium supplementation illustrates the principle of context-dependence: protective in deficient populations, but neutral or even harmful in selenium-replete cohorts (Rayman, 2020). This underscores the clinical necessity of integrating biochemical profiling into decision-making for supplementation.

The future of cardiovascular supplementation lies in its ability to complement guideline-directed medical therapies and address residual risk. Promising avenues include EPA in conjunction with statins to target triglyceride-driven risk, CoQ10 supplementation to enhance myocardial energetics in heart failure, and magnesium repletion to reduce arrhythmogenic potential in at-risk populations (Mortensen et al., 2014). Moreover, nutrient–nutrient interactions such as magnesium–vitamin D synergy or selenium–thyroid hormone interplay may further expand the therapeutic potential of supplementation. These integrative strategies require rigorous validation through large-scale, biomarker-enriched randomized controlled trials.

Despite generally favorable tolerability profiles, supplementation is not devoid of risk. High-dose omega-3 fatty acids have been associated with an increased incidence of atrial fibrillation, and co-administration of garlic or omega-3 with anticoagulants may increase bleeding risk (Nicholls et al., 2020; Kalstad et al., 2021). Regulatory oversight is therefore essential to ensure supplement purity, potency, and accurate labeling. Furthermore, international harmonization of upper intake levels for trace elements such as selenium would mitigate regional discrepancies and promote global safety standards.

The convergence of nutrigenomics, metabolomics, and digital health technologies offers a vision of truly personalized supplementation. Artificial intelligence-driven integration of genetic, metabolic, and lifestyle data could facilitate dynamic, patient-specific regimens, moving beyond empirical dosing toward adaptive precision care. Such advances will require interdisciplinary collaboration spanning cardiology, nutrition, genetics, and regulatory sciences, as well as novel trial designs capable of capturing synergistic nutrient–drug and nutrient–nutrient interactions over long-term follow-up.

## 8 Conclusion

Cardiovascular supplementation is evolving from a broad-based wellness concept into a mechanistically guided adjunct therapy. High-dose prescription EPA (icosapent ethyl) demonstrates robust evidence of MACE risk reduction in hypertriglyceridemic, high-risk populations, while mixed EPA + DHA supplements exhibit more variable efficacy. Coenzyme Q10—particularly in its ubiquinol form—improves myocardial bioenergetics and, potentially, heart failure outcomes, though inconclusive data in SAMS and inconsistent study designs warrant further investigation. Magnesium repletion remains essential for *bona fide* deficiency states, given its beneficial effects on vascular tone, arrhythmogenesis, and metabolic control, yet broad supplementation in the general population lacks strong supporting evidence. Selenium underscores the exigency of context-dependent supplementation; it prevents cardiomyopathy in deficient demographics but confers no proven advantage and may elevate risks if ingested in supraphysiologic doses.

Other supplements display more modest or heterogeneous findings. Vitamin D may benefit only those with clear biochemical deficiency; L-carnitine shows promise in specific conditions (e.g., peripheral arterial disease, dyslipidemia), pending comprehensive safety validations; and garlic extract yields modest improvements in blood pressure and lipids, generally serving best as an adjunct to conventional therapy.

Ultimately, a structured pathway—comprising biomarker assessment, rational formulation choice, biological response validation, and consistent endpoint monitoring—should govern the clinical use of cardiovascular supplements. Universal high-dose regimens are unlikely to be beneficial and may be detrimental; a precision-based, biomarker- and phenotype-driven approach offers the safest, most efficacious route forward. Such a paradigm shift can transition supplementation from empirical utility to evidence-based, mechanism-informed practice, delivering targeted advantages to patients who stand to gain the greatest benefit.

It is important to acknowledge a significant limitation in the current evidence base. The large-scale trials and meta-analyses cited herein predominantly originate from high-income countries. However, populations with the highest prevalence of nutritional deficiencies, and thus potentially the greatest absolute benefit from supplementation, often reside in low- and middle-income countries (LMICs). The translational impact of our conclusions is therefore constrained by this geographic research gap. Challenges such as healthcare infrastructure, cost of biomarker testing, and access to high-quality supplements further complicate the implementation of precision supplementation strategies in these settings. Future research must prioritize inclusive trial designs that enroll populations from diverse socioeconomic and geographic backgrounds to ensure equitable advancement in cardiovascular nutritional science and to develop feasible, context-specific interventions.
